# Regenerative Medicine Technologies to Treat Dental, Oral, and Craniofacial Defects

**DOI:** 10.3389/fbioe.2021.704048

**Published:** 2021-08-06

**Authors:** Jessica M. Latimer, Shogo Maekawa, Yao Yao, David T. Wu, Michael Chen, William V. Giannobile

**Affiliations:** ^1^Department of Oral Medicine, Infection, and Immunity, Harvard School of Dental Medicine, Boston, MA, United States; ^2^Department of Periodontology, Graduate School of Medical and Dental Sciences, Tokyo Medical and Dental University, Tokyo, Japan; ^3^Department of Periodontics & Oral Medicine, University of Michigan School of Dentistry, Ann Arbor, MI, United States; ^4^Biointerfaces Institute, University of Michigan, Ann Arbor, MI, United States; ^5^Laboratory for Cell and Tissue Engineering, Harvard John A. Paulson School of Engineering and Applied Sciences, Boston, MA, United States; ^6^Wyss Institute for Biologically Inspired Engineering, Harvard University, Boston, MA, United States

**Keywords:** bone regeneration, 3D printing, biocompatibility, regenerative medicine, tissue engineering, periodontal diseases/therapy, bioresorbable scaffolds

## Abstract

Additive manufacturing (AM) is the automated production of three-dimensional (3D) structures through successive layer-by-layer deposition of materials directed by computer-aided-design (CAD) software. While current clinical procedures that aim to reconstruct hard and soft tissue defects resulting from periodontal disease, congenital or acquired pathology, and maxillofacial trauma often utilize mass-produced biomaterials created for a variety of surgical indications, AM represents a paradigm shift in manufacturing at the individual patient level. Computer-aided systems employ algorithms to design customized, image-based scaffolds with high external shape complexity and spatial patterning of internal architecture guided by topology optimization. 3D bioprinting and surface modification techniques further enhance scaffold functionalization and osteogenic potential through the incorporation of viable cells, bioactive molecules, biomimetic materials and vectors for transgene expression within the layered architecture. These computational design features enable fabrication of tissue engineering constructs with highly tailored mechanical, structural, and biochemical properties for bone. This review examines key properties of scaffold design, bioresorbable bone scaffolds produced by AM processes, and clinical applications of these regenerative technologies. AM is transforming the field of personalized dental medicine and has great potential to improve regenerative outcomes in patient care.

## Introduction – Regenerative Medicine in Dentistry

### Etiology of Dental and Craniomaxillofacial Bone Deformities

Hard tissue deficiencies in the maxillofacial region are the result of numerous diseases, disorder and injuries, and appropriate rehabilitative therapies are necessary to restore quality-of-life for affected individuals. The Global Burden of Diseases, Injuries, and Risk Factors Study 2017 (GBD 2017) revealed that oral disorders had the greatest age-standardized prevalence and incidence in the world (Spencer et al., 2018). Periodontal disease is a significant contributor to oral disease burden; in 2017, the reported global prevalence was 796 million and the percentage change in age-standardized rates for this high impact disease has continued to increase (Spencer et al., 2018). Periodontitis is a chronic, multifactorial inflammatory disease associated with host-microbiome dysbiosis (Papapanou et al., 2018). The disease pathogenesis involves a complex, immunoinflammatory response, modulated by individual microbial, environmental, and genetic factors (Kornman, 2008). Further, periodontal disease is strongly interrelated with overall health, as evidenced by the vast number of oral manifestations in systemic diseases (Kornman et al., 2017; Albandar et al., 2018). Consequences of periodontitis include progressive deterioration of the periodontal attachment apparatus and alveolar bone, ultimately resulting in tooth loss and oral dysfunction (Page and Kornman, 1997; Pihlstrom et al., 2005). The disease may be further characterized by continuous progression, intermittent periods of disease activity (Goodson et al., 1982), or an “asynchronous multiple burst” model (Socransky et al., 1984), to which older adults are more susceptible (Page and Kornman, 1997; Marcenes et al., 2013).

Similar to trends for periodontal disease, incidence rates for cancers of the lip and oral cavity are also increasing (Spencer et al., 2018). Squamous cell carcinoma (SCC) is the leading form of head and neck cancer and the recent surge in prevalence is primarily attributed to oncogenic types of human papillomavirus (HPV) infection (Gillison et al., 2015; Menezes et al., 2021). High level evidence implicates HPV in a quarter of oral cavity cancers and well over half of cases in the oropharynx (Abogunrin et al., 2014; Rodrigo et al., 2014). Malignant tumors involving the oral cavity are often treated by surgical resection, accompanied by other treatment modalities such as radiotherapy, chemotherapy, or immunotherapy. Remission may be attained at the expense of a substantial loss of tissue and large residual bone defects in the maxillofacial region (Muzaffar et al., 2021). Another common cause of hard tissue deficiencies include craniomaxillofacial trauma resulting from motor vehicular collisions, falls, and other accidents (Manodh et al., 2016). According to the GBD 2017, head injuries had a global prevalence and incidence of 47 and 21.6 million, respectively (Spencer et al., 2018). Ultimately, craniomaxillofacial bone defects have a wide range of etiologies including infection, periodontal disease, oral cancer, tooth extraction or tooth loss, and trauma (Bodic et al., 2005). These bone deficiencies can detrimentally affect facial esthetics and important oral functions such as mastication, speech, and nutrition, thereby significantly impairing patient quality-of-life.

### Clinical Treatment of Periodontal Defects

The regeneration of periodontal defects in humans is case-sensitive due to the involvement of multiple tissue types and variability in defect morphology (Kao et al., 2015; Yu et al., 2019). For instance, a single defect in the periodontium may consist of all four of its major anatomical components: the gingiva, cementum, periodontal ligament (PDL), and alveolar bone (Smith et al., 2015). The regeneration of these tissues and their unique interfaces is necessary to restore full function as a supportive structure for the teeth (Melcher, 1976). Generally, vertical intrabony defects progress more rapidly than horizontal defects and are at an increased risk for tooth loss (Papapanou and Wennstrom, 1991). Moreover, a residual probing pocket depth (PPD) ≥ 7 mm after periodontal treatment represents risk for tooth loss at a 64.2 odds ratio compared to a PPD of ≤3 mm (Matuliene et al., 2008). In turn, tooth loss initiates anatomic remodeling processes which precede the formation of localized deficiencies in alveolar bone (Araujo et al., 2005). Considering the dramatic decrease in prognosis associated with defect progression and imminent ridge resorption after tooth loss, periodontal defects require timely intervention in order to maintain teeth and their associated bone volume.

The prognosis of regenerative periodontal therapy is dictated by the defect morphology, which primarily considers the number of remaining bone walls and the defect angle (Klein et al., 2001; Reynolds et al., 2015). 3-wall intrabony defects and class II furcations are well-contained spaces that offer the most predictable indications for periodontal regeneration. Defects with fewer bony walls or wider angles tend to be more difficult to treat and the results are often unpredictable (Klein et al., 2001; Reynolds et al., 2015). Other factors that decrease prognosis include an unfavorable vertical sub-classification of furcation involvement, root proximity and root concavities (Aichelmann-Reidy et al., 2015; Tonetti et al., 2017). Following complete debridement to reduce bacterial load and remove granulomatous tissue, periodontal regeneration can be achieved with or without biologics. In dental regenerative medicine, the most commonly used biologics are enamel matrix derivative (EMD) (Hammarstrom et al., 1997; Tsai et al., 2020) and recombinant human platelet-derived growth factor (rhPDGF-BB) (Nevins et al., 2003). rhPDGF-BB has demonstrated acceleration of clinical attachment level (CAL) gain and improved bone fill in the reconstruction of periodontal defects (Nevins et al., 2005; Nevins et al., 2013; Tavelli et al., 2021b). Recently, the second-generation platelet concentrate, platelet-rich fibrin (PRF), has attracted widespread attention for its regenerative potential in soft tissues, however, influence on bone healing and periodontal regeneration is not well established (Tsai et al., 2020). Also of note, clinical trials using fibroblast growth factor-2 (FGF2) have demonstrated promising results for the regeneration of periodontal defects (Cochran et al., 2016; Kitamura et al., 2016) and this biologic currently has approval for use in Japan.

**FIGURE 1:**
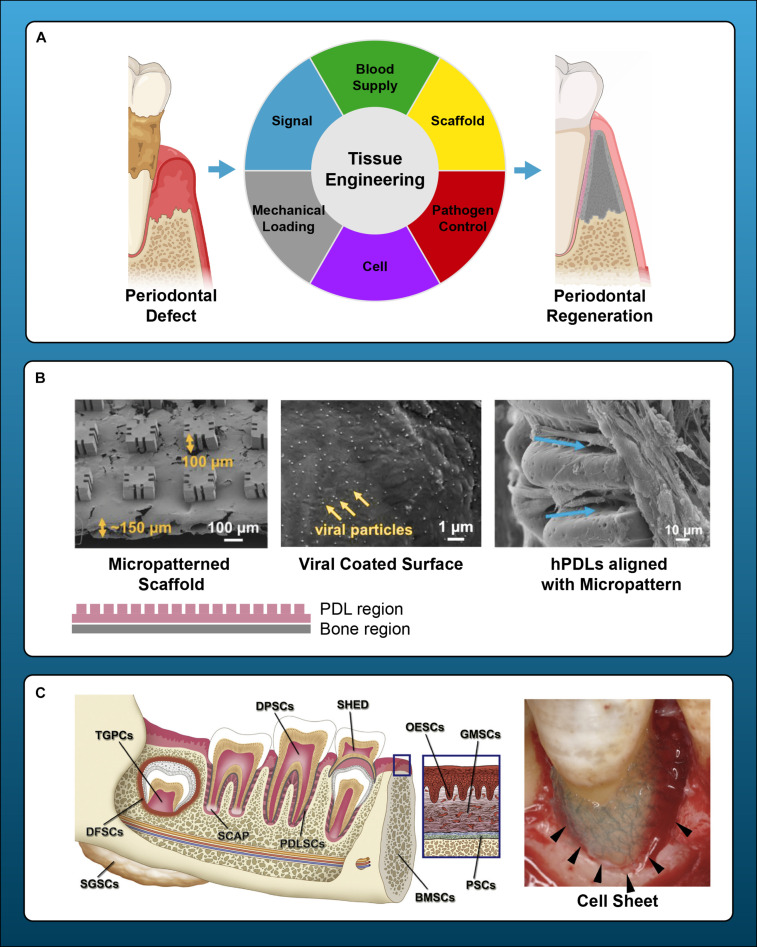
Principles and current endeavors for periodontal regeneration with tissue bioengineering. **(A)** Key components of periodontal regeneration with tissue engineering. Cells, growth factors, scaffold, mechanical loading, pathogen control, and ideal blood supply are the key for periodontal regeneration. **(B)** Examples of micropatterned scaffold, which enhances the orientation of fiber in periodontal regeneration. Left panel: SEM image of a micropatterned scaffold with grooves. Center: Viral Gene delivery (Ad-BMP-7) with chemical vapor deposition. Right: human PDL cells aligned along with the grooves of micropattern. **(C)** Left: prospective sources of stem cells in dental and maxillofacial region. BMSCs, bone marrow-derived mesenchymal stem cells from orofacial bone; DPSCs, dental pulp stem cells; SHED, stem cells from human exfoliated deciduous teeth; PDLSCs, periodontal ligament stem cells; DFSCs, dental follicle stem cells; TGPCs, tooth germ progenitor cells; SCAP, stem cells from the apical papilla; OESCs, oral epithelial progenitor/stem cells; GMSCs, gingiva-derived MSCs; PSCs, periosteum-derived stem cells; SGSCs, salivary gland-derived stem cells. Right: autologous PDL-derived a three-layered cell sheet with woven PGA. Adapted with permission from Egusa et al. (2012), Iwata et al. (2018), Pilipchuk et al. (2018), and Yu et al. (2019).

The key elements of periodontal regeneration are cells, scaffolds, growth factors, and blood supply (Larsson et al., 2016). Improved knowledge of how these components interact to promote periodontal tissue formation (Figure 1), accompanied by the advancement of microsurgical techniques and modern biomaterials, has led to the development of minimally invasive treatment approaches with improved clinical outcomes (Cortellini and Tonetti, 2011; Cortellini, 2012; Schincaglia et al., 2015; Moreno Rodriguez et al., 2019; Aslan et al., 2020; Barbato et al., 2020). While clinical standards for regeneration are usually well-achieved, true, histologic periodontal regeneration, involving formation of new cementum, PDL, and alveolar bone, remains elusive and instead, periodontal repair is often observed (Sculean et al., 2008). Animal studies have revealed that conventional guided tissue regeneration (GTR) results in long junctional epithelium and connective tissue (Sculean et al., 2015a), rather than an anatomic, periodontal attachment apparatus. In humans, EMD application paired with the coronally advanced flap (CAF) technique promoted new bone and cementum formation in the apical region of Miller class I and II (Miller, 1985) gingival recession defects (McGuire et al., 2016). Overall, periodontal regeneration requires technical surgeries and judicious, decision-making strategies to adapt a broad range of biomaterials, either in combination or alone, to achieve desired biologic and clinical results (Tavelli et al., 2020).

### Clinical Approaches in the Treatment of Alveolar Ridge Deficiencies

Dental implant therapy is often the treatment of choice to replace missing teeth, offering patients high satisfaction and improved oral health-related quality-of-life following treatment (Feine et al., 2018). The suitability of an edentulous site for implant placement is contingent on a sufficient, available bone volume (Avila-Ortiz et al., 2014). With advanced computer-aided design (CAD), virtual planning of the restorative position can accurately guide preoperative assessments of the residual ridge. Alveolar ridge augmentation with hard and soft tissue is frequently required to support a functional and esthetic result. In larger defects, guided bone regeneration (GBR) using barrier membranes and bone grafts may be performed, followed by implant placement and peri-implant soft tissue phenotype modification, if indicated (Tavelli et al., 2021a). Mounting evidence supports the augmentation of the peri-implant soft tissue volume and keratinized mucosa width to promote peri-implant health and stability of the marginal bone level (Giannobile et al., 2018; Longoni et al., 2019; Tavelli et al., 2021a). Autologous grafts remain the most effective treatment for soft tissue augmentation (Zucchelli et al., 2020). However, xenografts may offer similar clinical results with improved patient-reported outcomes in terms of pain and satisfaction (McGuire et al., 2020). With regards to hard tissue augmentation, autogenous bone grafts impart osteogenic influence and are often considered as the “Gold Standard” for regeneration (Al-Moraissi et al., 2020). Their disadvantage is that large quantities of graft material necessitate a secondary surgical site, such as the mandibular ramus or symphysis, in which donor-site morbidity and limited available bone volume for harvest are important considerations (Sculean et al., 2015b). As GBR requires substantial amounts of bone graft material compared to periodontal defects, a mixture of autograft and xenograft is commonly used (Urban et al., 2011, 2013).

Alveolar ridge deficiencies are categorized by their severity and defect type, generally described as horizontal, vertical, or combined (Seibert, 1983; Allen et al., 1985; Seibert and Salama, 1996; Wang and Al-Shammari, 2002). More severe and combined defects may require multiple surgical procedures for augmentation and are difficult to regenerate. Reported survival rates of dental implants placed in resultant bone from GBR procedures is comparable to rates in native bone (Jensen and Terheyden, 2009; Clementini et al., 2012). According to a recent systematic review, weighted means of clinical vertical bone gain were 8.04 mm for distraction osteogenesis, 4.18 mm for GBR, and 3.46 mm for bone block grafts, and post-operative complication rates were 47.3, 12.1, and 23.9%, respectively (Urban et al., 2019). GBR is technique-sensitive as surgical success relies upon adequate flap release to achieve primary closure and proper membrane application to prevent ingrowth of connective tissues into the bone compartment (Eskan et al., 2017; Soldatos et al., 2017). Non-resorbable membrane exposure, which is the predominant post-operative complication, occurs at rates of 13.8% in horizontal augmentation and 18% in vertical augmentation (Jensen and Terheyden, 2009). The development of dense, polytetrafluoroethylene (PTFE) membranes has enabled ridge preservation without primary closure, facilitating comparable results to GBR using e-PTFE membranes while reducing complications (Urban et al., 2019).

### Current Research Gaps

The clinical limitations, implications of invasive reconstructive surgical procedures, and prognostic uncertainty are current challenges in regenerative dental medicine. Clinical scenarios in which predictable treatments have yet to be achieved include ridge defects with severe horizontal or vertical components of alveolar bone loss, class III furcations, papilla deficiencies, and advanced peri-implant defects (McGuire and Scheyer, 2007; Reynolds et al., 2015; Monje et al., 2019). Additionally, few clinical strategies emphasize bone regeneration in the craniofacial complex. Defects in the calvaria, facial bones, and temporomandibular joint (TMJ) are often reconstructed with customized metal plates and implants with varying degrees of success. However, anatomic regeneration of functional craniomaxillofacial bone structures has yet to be achieved (Zhang and Yelick, 2018). Current regenerative biomaterials for bone commonly present issues related to early resorption or persistence, and limited capacity to reconstruct large or uncontained defects (Giannobile et al., 2019; Tao et al., 2019). Since 2000, regenerative medicine research, mainly in the field of bioengineering, has made significant progress. A broad range of research has been conducted using stem cells (Kaigler et al., 2013, 2015; Iwata et al., 2018; Xuan et al., 2018; Park J. Y. et al., 2020; Sanchez et al., 2020), gene delivery (Jin et al., 2003; Dunn et al., 2005; Chang et al., 2009, 2010; Sugano et al., 2014; Zhang Y. et al., 2015), surface modification with microstructures (Pilipchuk et al., 2016; Zhang Z. et al., 2016; Pilipchuk et al., 2018), three-dimensional (3D) bioprinting (Rasperini et al., 2015; Raveendran et al., 2019), and whole tooth regeneration (Kim et al., 2010; Oshima et al., 2011, 2017; Oshima and Tsuji, 2015). Additionally, clinical trials of microstructure-applied scaffolds (Rasperini et al., 2015; Raveendran et al., 2019) and PDL-derived cell sheets or PDL-derived mesenchymal stem cells (MSCs) have been conducted in humans (Iwata et al., 2018; Sanchez et al., 2020). The clinical regeneration of oral, dental, and craniofacial structures has advanced tremendously in recent years but there are still considerable needs for improving the customization of scaffolds to complex architectures to gain more predictable outcomes. Usage of modern scaffold fabrication techniques in coordination with biologic agents and novel cellular and molecular therapies are expected to develop the next generation of biomaterials in bone tissue engineering.

## Key Properties of Scaffold Design

### Biomaterial Compatibility With the Manufacturing Process

Material selection is critical in the design of scaffolds produced by additive manufacturing (AM) techniques. Suitable biomaterials must demonstrate process compatibility with the specific AM technique applied, as well as appropriate biochemical and physical characteristics to function successfully *in vivo* (Bourell et al., 2017). Although optimal processing parameters vary between the different forms of AM, typical features of a suitable material include buildability of the incrementally deposited layers, adequate densification after chemical or thermal treatments, and structural tolerance of other post-processing steps (Gu, 2015). Certain combinations of materials and AM processes may not facilitate adequate process accuracy, thus detrimentally affecting the consistency of a scaffold’s internal architecture, overall part quality, and reproducibility (Leong et al., 2003). Additionally, techniques involving processing steps that employ high temperatures (Han et al., 2017; Ligon et al., 2017), ultraviolet light irradiation (Bagheri and Jin, 2019), or organic solvents (Mikos and Temenoff, 2000) may preclude the simultaneous incorporation of cells and other biological factors.

AM technology enables scaffold production with a diverse array of materials, including polymers, metals, ceramics, hydrogels, and carbon-based nanomaterials (Guvendiren et al., 2016). Thermoplastic polymers are often used in extrusion-based technologies whereas ceramic, metal, or polymer powders are typically processed at higher temperatures in laser-based methods (Zhang S. et al., 2015; Yang et al., 2019). Recently, biodegradable metal alloys containing magnesium (Mg) or Zinc (Zn) are of increasing interest due to their improved corrosion resistance and biomimicry (Li et al., 2018; Wen et al., 2018; Hernández-Escobar et al., 2019). However, these materials present unique challenges such as high melting points, flammability, and generation of metallic vapors that compromise process stability (Grasso et al., 2018). Numerous synthetic polymers are practical material choices for AM fabrication of biomedical implants due to their high biocompatibility, biodegradability, bioresorption, and processability (Puppi et al., 2010). Polycaprolactone (PCL) is the most commonly used biomaterial in AM due to its excellent mechanical properties, low cost, ease of processability, and low melting point.

While polymers are excellent materials in their ability to accommodate AM processing parameters, singular material groups are limited in their capacity to mutually satisfy requirements for both AM processing and clinical utility in biomedical applications. For instance, polylactides have high tensile strength accompanied by a slow degradation rate, which may persist longer than desirable *in vivo*. In contrast, although polyglycolic acid (PGA) and poly lactic-co glycolic acid (PLGA) offer superior mechanical properties, they degrade quickly when used as a bioresorbable scaffold and within 2 weeks, their tensile strengthen is reduced by half (Ikada, 2006). To address this obstacle, ceramics are often combined with polymers to form composite materials with improved mechanical characteristics and biologic properties (Nyberg et al., 2017; Zhang et al., 2018).

Bone itself is a composite tissue by nature, consisting of a mineral phase predominated by nanocrystalline hydroxyapatite (HA) and an organic phase, consisting of extracellular matrix proteins, of which approximately 90% is collagen type 1 (Paschalis et al., 2003). The presence of mineralized collagen fibers affords bone both high elasticity and strength to prevent fracture during weight-bearing activities (Nair et al., 2013). In the periodontium, alveolar bone houses the dentition in fibrous joints classified as gomphoses. The tooth-bone interface is mediated by the PDL, a well-vascularized structure constituted by collagenous sheets of extracellular matrix, extending from alveolar bone and embedding into the root cementum (Naveh et al., 2013). Fibers of the PDL exhibit region-specific orientation that participate in physiologic loading, nutrient transport, and bone remodeling (Connizzo et al., 2021). Due to complex organization and composition required for function, multi-material constructs have superior capability to replicate hybrid tissue structures and promote scaffold performance (Jakus and Shah, 2017; Kim et al., 2018).

### Tailored Biomechanical Properties of Materials for Use in Alveolar Bone Reconstruction

By mimicking the physiologic characteristics of native bone, material property tailoring enhances the regenerative capacity of tissue engineered constructs in the presence of biomechanical stresses (Palmer et al., 2008). This is especially relevant for dental applications as the bone that comprises the periodontium and jaws is regularly subject to extrinsic forces (Korioth et al., 1992) that result in a physiologic degree of elastic deformation (Daegling et al., 1992; Korioth and Hannam, 1994). Consequently, alveolar bone is anisotropic in nature, meaning that it demonstrates a non-linear, elastic symmetry (Giesen et al., 2001; Peterson et al., 2006). This regional and directional variation in modulus is imparted by the structural orientation of mineralized collagen fibers and aids proper stress distribution (Lettry et al., 2003; Wang and Ural, 2018). The elastic modulus of trabecular and cortical bone have been reported to be in the ranges of 3.5–125.6 MPa (Misch et al., 1999) and 6.9–16.0 GPa, respectively (Dechow et al., 2010). AM techniques can produce versatile scaffolds with mechanical properties within these physiologic ranges for craniofacial and dentoalveolar reconstruction. This has been demonstrated in degradable polymers, calcium phosphate ceramics, and composite ceramic-polymer scaffolds fabricated with both direct and indirect means of solid free-form fabrication (SFF) (Hollister et al., 2005).

The layered construction process utilized in AM is advantageous for the production of lightweight and porous constructs that can support tissue regeneration in an irregular defect. CAD files can be used to generate scaffold configurations that accurately replicate the overall defect shape and dimensions. Further, customized 3D surface topology can be generated by using standard triangle language (STL) files to topologically subtract defects from a digital scaffold design (Park et al., 2012). This promotes anatomical scaffold adaptation to the defect boundaries, minimizing dead space and micromotion (Grottkau et al., 2002). An effective scaffold should also provide sufficient rigidity to sustain matrix deposition until newly formed tissue has developed the mechanical integrity to withstand normal load bearing conditions. Resistance to deformation is largely dictated by material selection, degradation rate, internal geometry and porosity. Cell seeding can further reinforce scaffolds through enhanced extracellular matrix production while also compensating for the gradual decline in structural integrity that accompanies degradation (Spalazzi et al., 2006a). Finally, modulus matching of the scaffold material to bone is essential to prevent disadvantageous mechanoregulation of anatomic remodeling (Sandino and Lacroix, 2011), as well as other adverse sequelae, such as scaffold fragmentation and stress shielding. Stress shielding occurs when an implanted substrate has a higher modulus than the surrounding host bone, creating areas of differential strain distribution on the adjacent tissue (Orr et al., 2001) and resulting in a localized decrease in density of the surrounding bone (Spector, 1994; Van Lenthe et al., 1997).

Mechanical cues provided by scaffold materials can regulate the fate of stem and progenitor cells (Vining and Mooney, 2017). In 2D culture, mechanical properties of the extracellular matrix (ECM) such as stiffness dictate the differentiation of MSCs derived from bone marrow or adipose tissues (Engler et al., 2006). In 3D systems, Huebsch et al. (2010) showed that the elasticity of the substrate or matrix appears to direct MSCs differentiation to the cell fate that best matches the elasticity of the native physiological ECM; stiffer matrix (11–30 kPa) stimulates osteogenic differentiation while softer matrix (2.5–5 kPa) promotes adipogenic or neuronal differentiation. Furthermore, variations in matrix stiffness can regulate MSC behaviors such as cell fate and migration (Tse and Engler, 2011). MSCs may be more responsive to a gradient of stiffness established by tunable levels of crosslinking along a spatial axis in a hydrogel scaffold (Sunyer et al., 2016).

Viscoelasticity, a key property of living tissues, is another regulator of MSC behavior. Viscoelastic materials exhibit a combination of storage of elastic energy as a solid, and loss of mechanical energy as a fluid. These materials exhibit stress relaxation and hysteresis in the stress-strain relationship during loading and unloading. When a mechanical load is applied then removed, viscoelastic materials can dissipate energy (Chaudhuri et al., 2020). Chaudhuri et al. (2016) demonstrated that MSC cell fate and activity is regulated by tuning the stress relaxation of the alginate hydrogel scaffold, independently of the hydrogel’s initial elastic modulus, degradation and cell-adhesion ligand density. More specifically, MSC cell spreading, proliferation, and osteogenic differentiation, and bone matrix production are enhanced when encapsulated in hydrogels with faster stress relaxation. When implanting alginate hydrogels with tunable stress relaxation to deliver human MSCs into rodent calvaria defects, animals receiving fast-relaxing hydrogels showed significantly enhanced new bone growth, extensive matrix remodeling and hydrogel disappearance compared to the group that received slow relaxing, stiffness-matched hydrogels (Darnell et al., 2017).

Mechanistically, the effect of scaffold mechanics is mediated by adhesion-ligand binding via integrin, actin-myosin contractility and activation of mechanosensing and mechanotransduction pathways. For instance, matrix elasticity directed stem cell lineage specification is non-muscle myosin II dependent (Engler et al., 2006). In addition, when stiffness matched, stress relaxation led to increased nuclear translocation of the YAP transcription factor, a key transcription factor mediating mechanotransduction (Chaudhuri et al., 2016). In tissue engineering and regenerative medicine, synthetic matrices with defined mechanical and biophysical properties are useful to guide stem cells *ex vivo* prior to transplantation, and to tune stem cell behavior *in vivo* following transplantation in order to improve their regenerative capacity (Huebsch et al., 2015; Darnell et al., 2017; Vining and Mooney, 2017).

### Architectural and Topographical Determinants of Cell-Scaffold Interactions

**FIGURE 2:**
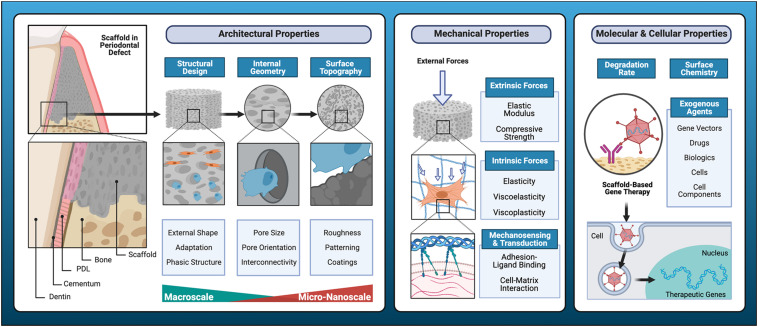
Key determinants of cell-scaffold interactions. Resorbable scaffolds for the regeneration of functional dental, oral, and craniofacial tissues require tailored, biomimetic features that consider structural design, internal geometry, and surface topography to promote cell-scaffold interactions. Additive manufacturing facilitates optimization of physical properties of scaffold substrates to promote overall mechanical performance and fine tune biomechanical regulation of cell behavior. Intrinsic material properties such as degradation rate and surface chemistry are key biochemical considerations, and various exogenous agents with bioactive properties may be incorporated for scaffold functionalization to further enhance regenerative outcomes.

AM can be used to produce sophisticated scaffolds with optimized macroscale architecture, internal geometry, and topographical features that enhance the requisite cellular processes for new tissue formation (Figure 2). The precise control of scaffold design afforded by AM techniques is a valuable feature for dental and craniofacial bone applications, as defects in these regions often involve multiple tissues that require complex spatiotemporal regulation for development (Lee et al., 2016). This presents the unique challenge of guiding the differentiation and maintenance of multiple, cellular phenotypes, as well as achieving synthesis of distinct but continuous tissues in a single construct. Triphasic scaffolds consisting of stratified compartments with unique material compositions mimic the organization of native tissues and enable tri-culture of chondrocytes, fibroblasts, and osteoblasts (Spalazzi et al., 2006b). This scaffold architecture efficaciously mediates phase-specific cellular proliferation and phenotypic matrix production (Spalazzi et al., 2006a, 2008).

Modern AM techniques have driven the evolution of hybrid scaffold systems designed for the regeneration of fibrous articulations within the craniofacial complex (Vaquette et al., 2018). 3D printed wax molds have been used to indirectly fabricate polymeric scaffolds with fiber-guiding microchannels to align fibroblasts and their subsequent connective tissue formation in a novel tooth to PDL interface (Park et al., 2010). Fused deposition modeling (FDM) and electrospinning techniques have been combined to produce biphasic periodontal scaffolds with well-integrated compartments for PDL and bone (Vaquette et al., 2012; Costa et al., 2014). Electrospinning methods have also produced functionally graded scaffolds with seamless transition zones (Erisken et al., 2008) and gradients in scaffold features such as pore size (Abbasi et al., 2019; Zhou et al., 2020).

It has been well established that pore characteristics mediate important cell-scaffold interactions that dictate cell morphology, phenotype maintenance, and biosynthetic activity (Nehrer et al., 1997). The recommended pore size for bone scaffolds ranges from 300 to 800 μm (Ishaug et al., 1997; Tsuruga et al., 1997), with the optimal size depending on the selected biomaterial composition and intended regional application of the scaffold. Larger pores are thought to facilitate vascularization, oxygenation, and direct osteogenesis while smaller pores may favor osteochondral ossification (Karageorgiou and Kaplan, 2005). Although the significance of pore size within this range may be minimal (Roosa et al., 2010), it is universally acknowledged that pores less than 100 μm in size prevent cellular infiltration and result in the formation of undesirable, non-mineralized osteoid or fibrous tissue (Hulbert et al., 1970). Further, small pores <125 μm in diameter prevent differentiation of MSCs (Swanson et al., 2021). As long as the selected pore size permits adequate cell migration for tissue ingrowth and osteogenic cell phenotypes, other features affecting fluid conductance (Hui et al., 1996), such as pore interconnectivity and orientation may be of greater influence. While conventional, porogen-leached scaffolds exhibit variable microarchitecture consisting of random interconnections, indirect SFF allows for controlled introduction of porosity, pore interconnectivity, and surface topography at the microscale (Taboas et al., 2003), resulting in superior distribution and quality of mineralized tissue formation *in vivo* (Park et al., 2012, 2014).

With increasingly advanced AM processes, high resolution features can be incorporated into the internal microarchitecture of a scaffold. Modern image rendering technology can develop biomimetic surface topographies that positively influence osteoblast behavior and local production of osteogenic factors such as osteocalcin (OCN), vascular endothelial growth factor (VEGF), osteoprotegerin (OPG), and bone morphogenetic protein (BMP)-2 (Cheng et al., 2016). Computer-directed deposition can produce micropores with customized orientation and interconnecting channels (Lim et al., 2010; Park et al., 2017). These features improve nutrient and oxygen diffusion throughout larger defect volumes and may also play a role in cell–cell communication. Microscale features such as patterning and surface roughness further enhance cell migration, adhesion, proliferation, and osteogenic differentiation (Zhu et al., 2020). This topographical influence on cell response is derived from effects on surface energy and protein adsorption, interactions that are recapitulated on the submicron (Wang et al., 2015) and nanoscales (Khang et al., 2012). Additionally, nanotopographical features can upregulate expression of genes known to be important for osteoblast adhesion, such as intercellular adhesion molecule 1 (ICAM1), integrin αM (ITGAM), integrin α1 (ITGA1) (Dalby et al., 2007), integrin α5 (ITGA5) and integrin β1 (ITGB1) (Liu et al., 2017).

### Scaffold Functionalization With Bioactive Molecules

Regenerative medicine is based upon the manipulation of known physiologic processes to create a microenvironment that simulates a desired stage of tissue development, thus inducing tissue formation and renewal. Not only must tissue dynamics be replicated at a macroscopic organ or tissue scale, but on the cellular and molecular levels as well. Scaffolds primarily serve to provide an osteoconductive matrix and benefit from the addition of growth factors that exert osteoinductive influence on cellular activity. Biologics such as recombinant human BMP-2 and BMP-7, growth differential factor-5 (GDF-5), EMD, and rhPDGF-BB, have all been well-studied for their capacities to promote osteogenic differentiation and enhance bone formation in regenerative dental medicine (Suárez-López Del Amo et al., 2015). Growth factor delivery strategies frequently take advantage of bioresorbable, polymer-based scaffolds as a carrier (Schliephake, 2010). The simplest method is scaffold immersion in a growth factor solution. However, drawbacks of physical adsorption include an initial burst release in the first 24 h followed by rapid attenuation (Caballé-Serrano et al., 2019). Post-processing, polyelectrolyte nanolayer coatings can deliver physiologically relevant quantities of active biologics with tunable release, however, this modification reduces pore area (Wei et al., 2007; Jin et al., 2008; Shah et al., 2014). Common strategies to simultaneously incorporate growth factors during the AM process include physical entrapment, which involves direct-loading of growth factor solution into structural reservoirs with a multi-head deposition system, or pre-loading, using a growth factor loaded paste as raw material in extrusion-based printing. In a study that compared these approaches, direct-loading exhibited similar issues to adsorption, in that it had diminished capability for sustained growth factor release (Huang et al., 2018).

Limitations in conventional growth factor delivery may be mitigated by the use of gene-activated scaffolds, in which a scaffold is utilized as a gene delivery device to facilitate controlled gene transduction upon implantation (Fang et al., 1996; Jin et al., 2004). Common methods of vector-based gene delivery may utilize peptides, viruses (adenovirus, baculovirus, or lentivirus), or non-viral vectors to deliver genes that induce expression of growth factors (Yan et al., 2019). Gene therapy has been further enhanced with treatments such as chemical vapor deposition (CVD) polymerization (Lahann et al., 2001), in which antibodies conjugated to adenoviral vectors for transgene expression (Hao et al., 2016) are immobilized onto a scaffold surface. This delivery mechanism allows for multi-growth factor gene expression to promote regenerative activities in target cells, as previously demonstrated with rhPDGF-BB and BMP-7 in human PDL fibroblasts (Hao et al., 2016). The same CVD-mediated, adenoviral vector treatment using adPDGF-BB and adBMP-7 was assessed in micropatterned, biphasic PLGA/PCL scaffolds implanted into alveolar bone defects *in vivo* (Pilipchuk et al., 2018). The results confirmed the ability to control the localization of multiple growth factors within a single scaffold construct to improve the formation and quality of regenerated periodontal tissues. Gene expression may be further altered by leveraging the epigenetic capabilities of microRNA (miRNA). MiRNA are small non-coding RNAs that regulate post-transcriptional modifications of a target messenger RNA and can inhibit translation of multiple genes by sequence-pairing homology (Larsson et al., 2015). Epigenetic functionalization of scaffolds to impart anti-inflammatory, immunomodulatory, or osteogenic influence may be achieved through incorporation of miRNA-transfected cells or direct loading of miRNA into the biomaterials (Asa’ad et al., 2020).

Finally, AM offers great capability to produce bioresorbable, scaffold-based drug delivery systems, incorporating pharmacologic agents that confer antimicrobial properties or other therapeutic effects. New bone formation occurs slowly over a period of several months and the scaffold material must persist for a relatively long duration of time. Biofilm colonization, localized tissue infection, and chronic inflammatory processes pose serious risks to the final regenerative outcome. Scaffolds produced by AM processes may address these concerns through the addition of antibiotics and corticosteroids, while enhancing the regenerative outcome. A 0.5 mg/ml concentration of doxycycline has been demonstrated to promote osteoblast differentiation *in vitro* (Almazin et al., 2009) and other research has identified anti-osteoclastic (Bettany et al., 2000) and anti-collagenase (Holmes et al., 2004) activity. It should be noted that pre-loading methods involving high temperatures can significantly reduce the efficacy of specific types of antibiotic compounds (Shim et al., 2015). Additionally, controlled release is essential, otherwise excessive dosages of antibiotics will confer cytotoxic effects on osteogenic cell populations (Feng et al., 2010; Park, 2011). Use of nanocoatings or nanofiber delivery mechanisms (Han et al., 2017; Li Z. et al., 2020) to convey antimicrobial properties should be further explored (Kumar et al., 2020). Scaffold modifications with anti-fouling, zwitterionic polymer coatings (Chen et al., 2019) and antimicrobial peptides (Liang et al., 2021) represent promising alternative strategies to discourage biofilm formation or microbial infections.

## Additive Manufacturing for Bone Tissue Engineering

### Paradigm Shift in Scaffold Production for Regenerative Medicine

AM is a layer-by-layer construction process used to create 3D constructs with CAD and computer-aided manufacturing (CAM) technology. It is often used interchangeably with SFF, which also implies the use of a fixture-less platform without part-specific tooling or human intervention, or rapid prototyping (RP), often used in contexts that describe fast fabrication of scale models or parts. AM processes first emerged in the 1980’s and have rapidly evolved to become a powerful tool for biomedical scaffold design, capturing particular interest from the research community in bone tissue engineering. Research involving bone-related applications account for approximately 20% of the existing publications in searches for articles with the terms “additive manufacturing” and “3D printing.”

AM fabrication of personalized biomedical constructs begins with the acquisition of high resolution, 3D datasets, typically with computed tomography (CT) or magnetic resonance imaging (MRI), from an individual patient. The images are then converted to a common medical file format referred to as digital imaging and communication in medicine (DICOM). The DICOM file is then imported into a software package that performs segmentation to produce serial sectional data (slices) and reconstruction of a volumetric model composed of voxels. A voxel is a single unit of volume within a 3D grid as opposed to a 2D pixel. A density threshold is then established and the signal intensity of point data is used to determine which points will be included. Surface polygons are then extracted to reconstruct a tessellated, polyhedral model, also known as a mesh which can be exported as an STL file. Surface refinement is performed with algorithms such as non-uniform rational B-spline (NURBS) functions; this can occur either before the creation of the STL-triangulated surface, known as reverse modeling or after, which is the STL-triangulated model converting approach (Sun et al., 2005). With these steps, a CAD-based solid model is available for further optimization and digital manipulation.

**FIGURE 3:**
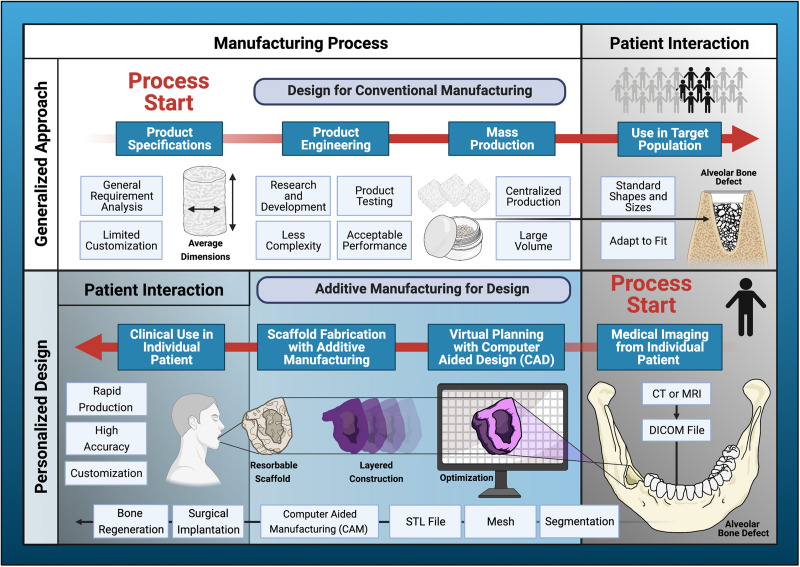
Paradigm shift in scaffold production. Additive manufacturing has introduced a departure from design for conventional manufacturing processes to additive manufacturing driven by design for the individual patient. The generalized design approach utilizes traditional product specification and engineering processes to facilitate large-scale production for distribution to a target population. Disadvantages of conventional manufacturing include limited capacity for complex designs and less customization. Additive manufacturing (AM) utilizes individual patient data processed by computer-aided design (CAD)/computer-aided manufacturing (CAM) software to perform virtual planning, design optimization, and fabrication of highly personalized scaffolds for bone regeneration. This design process begins and ends with direct patient interaction. AM has enormous potential to improve accessibility to personalized regenerative medicine in everyday clinical dentistry.

Dentistry is a field that has embraced AM techniques and frequently uses commercially available equipment to fabricate patient-specific constructs in everyday practice. AM is considered a technologic hallmark of the 4th industrial revolution and has resulted in a paradigm shift from design for manufacturing to manufacturing for design (Figure 3). In the next decade, AM is expected to drastically reduce the utilization of conventional manufacturing techniques and consequently transform employment dynamics in numerous industries (Pérez-Pérez et al., 2018). AM will give rise to new fields and technical occupations, as it has already done with the advent of computer-aided tissue engineering (Sun et al., 2005), and eliminate tasks that can be performed by automated processes. The future of clinical regenerative procedures will possibly involve biomedical laboratories staffed with bioengineers and computer technicians dedicated to the fabrication of personalized bone scaffolds, similar to the current scenario in which dental laboratories produce customized prosthetic components like dentures and crowns. While ongoing research has yet to produce a reliable AM protocol to create custom, bioresorbable scaffolds for bone reconstruction, this pending scientific development signifies untapped potential for unprecedented regenerative outcomes, as well as commercialization and economic growth.

### Types of Additive Manufacturing

**FIGURE 4:**
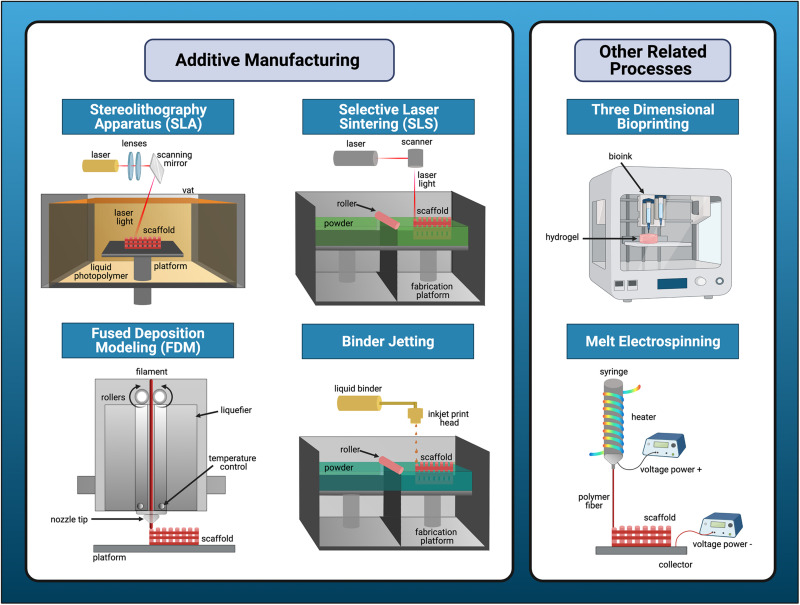
Overview of major types of additive manufacturing processes for bone tissue engineering applications. Additive manufacturing (AM) falls into three major categories: laser-based, extrusion-based, or binder jetting processes. Stereolithography apparatus (SLA) and selective laser sintering (SLS) are the predominant forms of laser-assisted techniques for production of non-metallic bone scaffolds. Fused deposition modeling (FDM) is the main extrusion-based method and binder-jetting is the last method. Melt electrospinning or bioprinting are similar, but distinct scaffold fabrication processes that may be used in conjunction with traditional methods of AM.

Depending on the specific tissue and critical defect size, there are numerous options for AM regenerative scaffolds in the oral and craniofacial arena. The predominant methods for non-metallic bone scaffold production can be categorized broadly into extrusion-based, laser-assisted, or binder jetting type processes (Figure 4). The details regarding the primary compatible materials and specific advantages and disadvantages of each AM technique is summarized in Table 1. The main extrusion-based method for non-metallic scaffold production is FDM. First developed in 1988, FDM is commonly used in the oral and craniofacial regeneration research areas. Materials are extruded as a filament through an output (nozzle or syringe) that is directed by CAD files obtained via radiology or similar imaging techniques (Mota et al., 2015). FDM’s main advantages include greater mechanical strengths and simpler processes relative to other AM techniques. When considering some of the complex oral internal structures, such as the intricate geometry of the periodontal ligament, the lack of resolution needed to create detailed features via FDM is a disadvantage when compared to other AM techniques (George et al., 2017). FDM enables high production rate at a low cost, which has positive implications for FDM’s ability to be used widely in the clinical setting. Additionally, FDM may be used in conjunction with other scaffold fabrication techniques such as electrospinning; preclinical research has shown potential of this combined approach for biphasic constructs employed in vertical bone augmentation (Sudheesh Kumar et al., 2018; Vaquette et al., 2021) and regeneration of the periodontal complex and supporting alveolar bone (Vaquette et al., 2012).

**TABLE 1:** Types of additive manufacturing processes and their general features.

AM technique	Process	Compatible materials	Advantages	Disadvantages
Fused deposition modeling (FDM)	Extrusion-based	PLA PCL β-TCP	High mechanical strength. No excess material inside scaffold.	Thermal processing. Low printing resolution (>100 μm).
Stereolithography apparatus (SLA)	Laser-assisted	HA CA	Cell and bioink carrier potential. Internal resolution.	Limited material diversity.
Selective laser sintering (SLS)	Laser-assisted	PCL PLA HA	No support structure necessary	Thermal processing
Three-dimensional printing (3DP)	Binder jetting	PCL	No heat or support structure necessary.	Low mechanical strength.
Melt electrospinning	Fiber-based	PCL	Tunable fiber thickness (<20 μm). High architectural control.	Limited material diversity.

*PLA, polylactic acid; PCL, polycaprolactone; TCP, tricalcium phosphate; HA, hydroxyapatite; CA, ceramic acrylate.*

Stereolithography apparatus (SLA) and selective laser sintering (SLS) are the primary laser-assisted techniques for non-metallic bone scaffold production. First developed in 1983, SLA utilizes photochemical reactions with UV lasers to produce scaffolds out of photosensitive polymers. Because of specific material and post-processing requirements associated with toxicity concerns, SLA is not as commonly used for craniofacial regeneration. The main advantage of SLA is its capability for high accuracy and refined internal resolution relative to other AM techniques (Msallem et al., 2020). First developed and then subsequently commercialized in 1992, SLS is a powder-based technique which utilizes a laser to sinter powder spread across a rolling plate. Scaffolds developed with SLS have strong mechanical properties suitable for bone and can be designed with complex geometries (Sudarmadji et al., 2011). SLS is especially useful for fabricating porous, bioactive bone scaffolds consisting of polymer-ceramic composites, most commonly involving the combination of HA and PCL (Xia et al., 2013; Du et al., 2015).

Melt electrospinning is a distinct processing technique often used in conjunction with AM. In general, this technique allows for the introduction of micro- and nano-scale features into regenerative scaffolds. The technique is similar to FDM, with the main difference being a high-voltage power supply to extrude precise droplets with a refined resolution. Electrospun fibers and scaffolds are particularly advantageous for drug or small molecule loading because of its nanoscale morphological structure (Chew et al., 2006). Biomaterials with antimicrobial properties offer a significant advantage in the regeneration of periodontal structures affected by periodontal disease, in which oral-biofilm is a key component in the dysregulated inflammatory response. An electrospun gelatin and low molecular weight chitosan scaffold demonstrated antimicrobial efficacy against *Aggregatibacter actinomycetemcomitans*, a facultative anaerobe commonly implicated in periodontal infections (Budai-Szűcs et al., 2021). Further, an ibuprofen-functionalized, nanofibrous PCL scaffold improved CAL and reduced expression of inflammatory mediators COX-2 and IL-8 in seeded human oral epithelial cells and fibroblasts challenged by *Porphyromonas gingivalis* lipopolysaccharide, a key pathogenic factor in periodontitis (Jain and Darveau, 2010). Alternatively, solution electrospinning involves polymer solutions and solvents to solubilize the solutions into materials in the scaffold design (Xue et al., 2019). This technique is still used with improved alignment, although melt electrospinning offers more detailed control over the architecture and less toxicity concerns for craniofacial regenerative purposes.

### Bioresorbable Scaffolds for Bone Reconstruction

**FIGURE 5:**
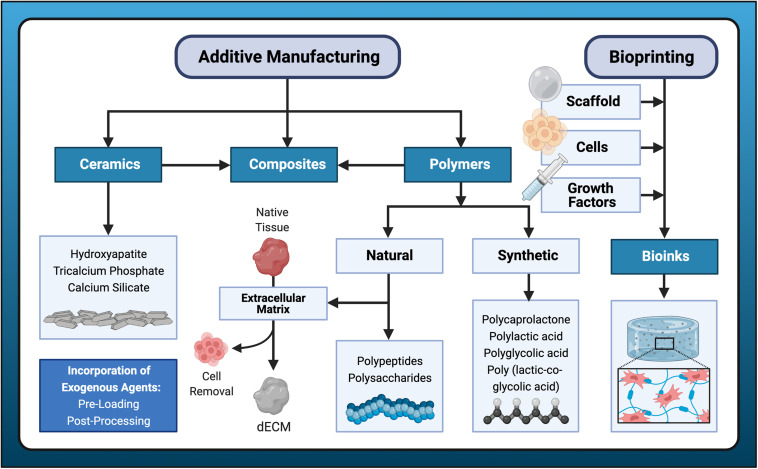
Biomaterials for bone scaffold fabrication. A variety of candidate materials are available for scaffold fabrication using additive manufacturing or bioprinting processes. Additive manufacturing typically employs polymers, to which ceramic materials may be added to form composites. Bioprinting incorporates all three elements of the tissue engineering triad: cells, scaffold (hydrogel), and growth factors. Exogenous agents are often incorporated either with pre-loading or post-processing methods.

Bioresorbable scaffolds are materials that may be degraded into moieties *in vivo*, undergoing subsequent elimination through natural pathways resulting in total removal of the initial material without adverse biologic effects (Vert et al., 1992). A large variety of bioresorbable materials with unique material properties and degradation rates are available for scaffold fabrication (Figure 5). The mechanism of degradation occurs either through highly specific enzymatic cleavage, as is the case for natural polymers such as collagen, or passive hydrolysis, which induces chain scission of synthetic polymers under physiologic conditions. The degradation rate is influenced by a multitude of factors including but not limited to the molecular weight, chain configuration, comonomer ratio, residual monomer content, and crystallinity, as well as annealing and sterilization procedures and incorporation of drugs or other additives (Yannas, 2015). Bioresorbable materials are advantageous in bone tissue engineering due to their ability to facilitate regeneration while eliminating the need for removal by a secondary surgical procedure. This is an essential feature for periodontal tissue regeneration, in which delicate connective tissue structures and their interfaces must be restored; removal of a non-resorbable material would traumatize the site and disrupt healing. A principal challenge in formulating bioresorbable materials is matching the degradation rate to the intrinsic pace of native tissue remodeling, while maintaining sufficient mechanical properties of the scaffold. Failure to do so poses a high risk of scaffold exposure in periodontal surgery due to inflammatory complications in the thin gingival tissues that overlay alveolar bone, however, this risk is also present in the use of non-resorbable, metallic scaffolds. Careful flap design and suturing technique must also be employed to obtain primary closure and promote normal wound healing.

Scaffolds for bone tissue engineering can be typically assigned to one of the following categories: natural biopolymers, synthetic polymers, ceramics, acellular tissue matrices, and composite materials composed of two or more material groups (Akter and Ibanez, 2016). Natural polymers are biologically active and can be further categorized into polypeptides or polysaccharides, which are both frequently used in 3D bioprinting techniques (covered in section “Three Dimensional Bioprinting”). Polypeptide-based materials in particular possess amino acid sequences associated with integrin-binding domains conducive to cell adhesion and growth (Filippi et al., 2020). Another notable advantage is their biodegradability, which facilitates host cell production of extracellular matrix to replace the degrading scaffold (Akter and Ibanez, 2016). Disadvantageous features of some natural materials include risk of immunogenicity, possibility for disease transmission, and relatively low mechanical strength.

Synthetic polymers are the largest group of biodegradable polymers and include poly(α-ester)s, polyurethanes, polyacetals, poly(ester amide)s, polyanhydrides, polyphosphazenes, and pseudo poly(amino acids) (Filippi et al., 2020). Their use are highly prevalent in AM techniques due to characteristic low melting points and versatile physical properties that accommodate a wide range of processing parameters. Due to their high biocompatibility, numerous synthetic polymers are FDA-approved and can be employed in a broad range of biomedical applications. The poly(α-ester) family is the most common bioresorbable material choice compatible with AM production of scaffolds for bone tissue engineering and includes PCL, PLGA, polylactic acid (PLA), and polyglycolic acid (PGA) (Burg, 2014). These polymers are also frequently combined with ceramic biomaterials, which not only enhance mechanical properties and osteoconductivity, but also confer osteoinductive and osteogenic potential due to their similar composition to the inorganic phase of bone (Ducheyne and Qiu, 1999; Chai et al., 2012). Incorporation of other bioactive compounds such as calcium-silicate, can improve polymer surface hydrophilicity (Lin et al., 2017) and provide osteostimulation (Zhai et al., 2017). Representative *in vivo* studies of bioresorbable polymeric and polymeric-composite scaffolds produced by AM techniques for bone regeneration are featured in Table 2.

**TABLE 2:** Representative *in vivo* studies using additive manufacturing (AM) to produce resorbable scaffolds for dental, oral, and craniofacial-related bone regeneration from 2010 to 2020.

Material	Added biologic components	AM method	Model; tissue types	Notable design features	Key outcomes	Illustration
PCL + β-TCP	Human osteoblasts** +** human PDLCs	Fused deposition modeling + electrospinning	Rat; periodontal complex	Biphasic scaffold with bone and PDL compartments combined with use of cell sheets.	The mixed-methods approach created well integrated but distinct compartments. Presence of cell sheets facilitated periodontal fiber attachment and cementum-like tissue.	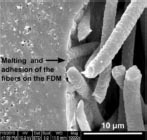 Vaquette et al., 2012
PCL + β-TCP	dECM from porcine bone** +** MC3T3 preosteoblast cells	Extrusion-based	Rabbit; calvaria	Composite polymer-ceramic material immersed in bone dECM solution.	Bone dECM imparted high quantities of BMP-2 and BMP-7 and enhanced MC3T3 differentiation *in vitro*. Bone volume fraction and bone mineral density was highest in PCL/β-TCP/dECM group *in vivo*.	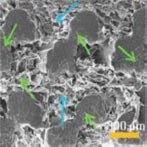 Kim et al., 2018
PCL + CS powder	dECM from MG64 cells	Extrusion-based	Rat; calvaria	dECM coating was applied to the scaffold to improve biocompatibility and cellular response.	CS/PCL/dECM improved cellular adhesion, proliferation, and differentiation of human MSCs, expression of osteogenic genes increased and pro-inflammatory genes decreased.	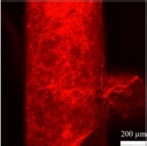 Wu et al., 2019
α-TCP powder + hardening liquid (5% sodium chondroitin sulfate, 12% disodium succinate, 83% distilled water).		3D inkjet printing	Human; maxilla, mandible, and frontal bone	Unsintered calcium phosphate was selected to promote replacement rate by native bone in large alloplastic grafts.	Satisfactory bone union occurred in 18 of 21 remaining sites at 1 year. Bone union was missing in the other three sites. Some host sites experienced resorption and no scaffolds underwent complete replacement.	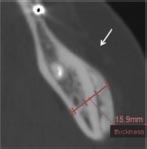 Kanno et al., 2016
Poly-ε -caprolactone + hydroxyapatite	SDF-1 + BMP-7	Extrusion-based	Rat; mandibular incisor	3D microstrands with interconnecting microchannels.	Orthotopic implantation showed tissue ingrowth and scaffold interface with fibrous tissue reminiscent of PDL and newly formed bone.	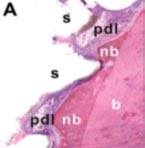 Kim et al., 2010
PCL	Human PDLCs + AdCMV-BMP-7	3D printing and indirect mold casting	Rat; periodontal complex	Controlled pore orientation and distinct tissue compartments with fiber-guiding channels.	Novel scaffold architecture directed spatial bone growth and enhanced bone volume fraction and tissue mineral density outcomes *in vivo* compared to control with random porous architecture.	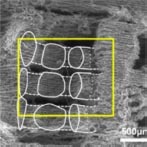 Park et al., 2012
PCL powder + 4% hydroxyapatite	rhPDGF-BB	Selective laser sintering	Human; periodontal complex	Internal port for growth factory delivery and fiber guiding pegs for periodontal ligament PDL orientation.	Initial 3 mm gain of clinical attachment and partial root coverage was achieved without inflammatory reaction at 12 months. Scaffold exposure occurred at 13 months due to slow degradation rate of PCL and ultimately necessitated removal.	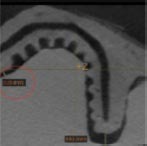 Rasperini et al., 2015
PLGA/PCL + amorphous PCL	AdPDGF-BB + AdBMP-7 + human PDLCs	Photolithography and indirect mold casting	Rat; periodontal complex	Micropatterned pillars and chemical vapor deposition to immobilize adenoviral gene vectors for PDGF-BB and BMP-7 expression.	Micropatterning promoted PDL maturation similar to the width of native PDL. Gene delivery groups showed increased expression of collagen III and periostin, as well as greater bone fill maintenance. Minimal cementum formation observed.	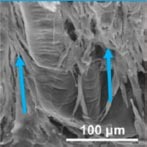 Pilipchuk et al., 2018

*PCL, polycaprolactone; TCP, tricalcium phosphate; PDLCs, periodontal ligament cells; dECM, decellularized extracellular matrix; BMP, bone morphogenetic protein; CS, calcium silicate; MSCs, mesenchymal stem cells; 3D, three-dimensional; SDF, stromal-derived factor; rhPDGF-BB, recombinant human platelet derived growth factor-BB; PLGA, poly lactic-co glycolic acid.*

Cell-derived, decellularized extracellular matrix (dECM) may also be combined with synthetic polymeric scaffolds to provide appropriate molecular cues for osteogenic activity. AM-printed constructs have been coated with dECM obtained from bone cells (Wu et al., 2019) or non-bone cells such as human lung fibroblasts (Kim et al., 2018) and MSCs from nasal inferior turbinate tissue (Pati et al., 2015). dECM has also been obtained from dental pulp (Sangkert et al., 2016). Preclinical experiments have demonstrated superior ability of dECM coatings to enhance new bone formation *in vivo* compared to bare scaffold controls (Pati et al., 2015; Kim et al., 2018; Wu et al., 2019). Further, dECM coatings downregulate expression of pro-inflammatory cytokines tumor necrosis factor α (TNF-α) and interleukin-1 (IL-1) and improve MSC adhesion, proliferation, and osteogenic differentiation through induction of attachment protein expression *in vitro* (Wu et al., 2019). Application of dECM coatings to scaffolds produced by AM addresses the need for balance between biologic and mechanical properties while overcoming limitations of tissue-derived ECM which consists of decellularized tissues or organs (Zhang W. et al., 2016).

### Three Dimensional Bioprinting

Bioprinting, generally defined as “the use of computer-aided transfer processes for patterning and assembly of living and non-living materials with a prescribed 2D or 3D organization to produce bioengineered structures” (Daly et al., 2021b), is a promising field in regenerative medicine, providing precise and controlled deposition of cells, hormones, drugs, and growth factors, etc. thus directing improved tissue regeneration (Aljohani et al., 2018). Among the broad range of 3D printing techniques, the most common and accessible bioprinting method is extrusion bioprinting, where the pressure-driven extrusion of a bioink from a printer head is used to print filaments following a defined design or pattern (Ozbolat and Hospodiuk, 2016). Inkjet printing falls under the umbrella of extrusion printing but involves the deposition of bioink droplets through the printhead rather than continuous filaments (Li X. et al., 2020). For extrusion-based or extrusion-related bioprinting, the bioink is a unique feature compared with cell-free 3D printing. Bioinks can generally be described as “a formulation of cells that is suitable to be processed by an automated biofabrication technology” (Groll et al., 2018), which usually has a hydrogel formulation as the main component containing cell-suspensions or cell aggregates (Daly et al., 2021b).

The selection of bioinks is one of the most critical steps in the process of bioprinting, mainly relying on two important aspects: biofabrication and biocompatibility. Biofabrication usually refers to the printability of the ink, such as the compatibility with the printer and printing resolution, which is highly related to the rheological properties of the bioink. Viscous and shear-thinning hydrogels, such as gelatin and methylcellulose (Ahlfeld et al., 2020), are often considered suitable for many bioprinting scenarios, as these materials can flow smoothly during extrusion, avoid the formation of clogging within the printhead, and stabilize after deposition. Biocompatibility involves the impact of the bioink on cell behaviors including short-term cell viability and long-term cell proliferation, migration, differentiation and organization. The cellular interactions with the bioink can be influenced by multiple factors simultaneously such as the gelation and deposition processes, as well as the biological and biophysical properties. Of note, a desired bioink might be specifically related to limited cell types and biological scenarios.

Dental, oral and craniofacial tissues are organized with complex 3D architectures involving multiple types of cells and tissues. Mimicking their 3D complexity and multicellular interactions represents one of the main barriers in dental and craniofacial regeneration (Obregon et al., 2015). 3D bioprinting holds great potential for creating 3D defect-specific constructs with multiple cell sources for use in regenerative medicine. 3D bioprinting studies applied to dental and craniofacial regeneration can be divided into three general focuses including the periodontal complex, pulp-dentin complex and craniomaxillofacial bone (Table 3). As periodontal ligament cells (PDLCs) contain stem cells that harbor the potential to generate cementum/PDL-like tissue (Seo et al., 2004), PDLCs are one of the most frequently-employed cell types for periodontal regeneration-oriented bioprinting. A previous study systematically investigated the printability of various concentrations of GelMA hydrogels and the influence of different 3DP parameters such as photoinitiator concentration, UV exposure, pressure and needle diameter on the viability of PDLCs in order to achieve high printing resolution, dimension ability and cell viability simultaneously for periodontal regeneration (Raveendran et al., 2019).

**TABLE 3:** Representative studies on 3D bioprinting for dental, oral, and craniofacial-related regeneration from 2016 to 2020.

Bioink	Bioprinting method	Tissue types	Cells/growth factors encapsulated	Key outcomes	Illustration
GelMA	Microextrusion-based	Periodontal complex	PDLCs	The optimized printing conditions supported a high level of PDLCs viability and facilitated cellular proliferation within the construct over 14 days.	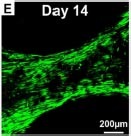 Raveendran et al., 2019
GelMA	Microextrusion-based	Pulp-dentin complex	hDPSCs + BMP-mimetic peptide	BMP-GelMA bioink formulation provided proper printability and dental specific microenvironment to support hDPSCs high viability, proliferation, and differentiation.	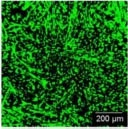 Park J. H. et al., 2020
Dentin-derived ECM + Alginate	Extrusion-based	Pulp-dentin complex	Odontoblast-like cell line (OD21) + acid-soluble dentin molecules	Dentin-derived ECM hybrid cell-laden hydrogel bioink showed high printability and cell survival. This hybrid hydrogel embedded with acid-soluble dentin molecules can enhance odontogenic differentiation.	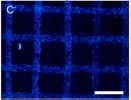 Athirasala et al., 2018
Fibrinogen + Gelatin + Hyaluronic acid + Glycerol	Custom-made syringe bioprinting	Whole tooth	hDPSCs	A dentin pulp complex with patient-specific shape was successfully produced by co-printing the bio-inks with polycaprolactone. After culturing for 15 days, localized differentiation of hDPSCs in the outer region of the construct was achieved with localized mineralization.	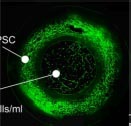 Han et al., 2019
ECM bioink (2% octapeptide) + AMP	Microvalve bioprinting	Craniomaxillofacial bone tissue	hDPSCs	The cell-laden bioprinted constructs modified with AMP exhibited a high level of mineralization and osteogenic gene expression *in vitro* and the ECM/1.0AMP composition displayed excellent bone regeneration capability *in vivo*.	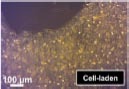 Dubey et al., 2020
Gelatin-alginate + cellulose nanofibrils + bioactive glass	Extrusion-based	Bone	(i) Human osteoblast-like cells (Saos-2). (ii) hBMSCs	The addition of bioactive glass and cellulose nanofibrils to gelatin–alginate system enhanced their printability and osteogenic activity but resulted in the death of Saos-2 cells due to increased viscosity.	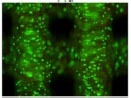 Ojansivu et al., 2019
GelMA + silicate nanoplatelets	Extrusion-based direct-writing bioprinting	Bone	HUVECs + hBMSCs + VEGF	Two GelMA hydrogels containing different concentrations of VEGF were optimized and bioprinted into well-defined 3D architectures, which resulted in the formation of a perfusable lumen, maturation of vascular vessels, and induced osteogenic differentiation.	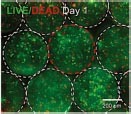 Byambaa et al., 2017
Agarose + collagen I	Inkjet	Bone	hBMSCs	Increased solids concentrations of collagen in the 3D-bioprinted hydrogel blend enhanced cell spreading, that ultimately contribute to enhanced and directed MSC osteogenic differentiation.	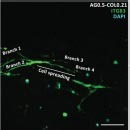 Duarte Campos et al., 2016

*GelMA, gelatin methacryloyl; PDLCs, periodontal ligament cells; hDPSCs, human dental pulp stem cells; ECM, extracellular matrix; AMP, amorphous magnesium phosphate; hBMSCs, human bone marrow-derived mesenchymal stem cells; HUVECs, human umbilical vein endothelial cells; VEGF, vascular endothelial growth factor; 3D, three-dimensional.*

The regeneration of pulp-dentin complex or the whole tooth has attracted great attention in dentistry. It is known that dental pulp stem cells (DPSCs) can differentiate into several cell types, including odontoblasts, neural progenitors, osteoblasts, chondrocytes, and adipocytes with high proliferative capability (Casagrande et al., 2011). Therefore, various studies have combined DPSCs with modified bioinks to establish 3D-bioprinted dental constructs. In previous research, a dentin matrix was isolated and combined with alginate to fabricate hydrogel blends as the bioink (Athirasala et al., 2018). The printability of the bioinks was greater in the formulations containing higher concentrations of alginate, whereas a higher proportion of dentin matrix proteins significantly improved cell viability and a 1:1 ratio of alginate and dentin was determined to be most suitable. Further, addition of acid-soluble dentin molecules into hydrogels enhanced odontogenic differentiation. Besides naturally derived molecules, synthetic biomolecules such as BMP-mimetic peptide have been incorporated into bioink as well. Park J. H. et al. (2020) developed a novel BMP peptide-tethering bioink formulation and found 50% of the peptides remained in the bioprinted construct after 3 weeks in an *in vitro* cell culture. The BMP peptide construct group exhibited the highest calcification as compared to the growth medium, osteogenic medium, and control groups with robust expression of osteogenic genes. In addition to pulp-dentin complex, the feasibility of whole tooth bioprinting has been studied by co-printing the hDPSCs-laden bioinks with PCL. The results not only achieved localized differentiation of hDPSCs in the outer region of the 3D cellular construct but also successfully produced 3D patient-specific cellular constructs for tooth tissue engineering in a predefined pattern (Han et al., 2019).

Engineering craniomaxillofacial bone tissue is a unique challenge due to the complex architecture of bone, consisting of organized calcified regions with interpenetrated vasculature (Salgado et al., 2004). In order to support osteogenesis, stem cells with osteogenic potential such as bone marrow-derived MSCs or DPSCs were frequently used. Moreover, various bioactive components have been incorporated into bioinks to enhance the osteogenic ability including amorphous magnesium phosphate (Dubey et al., 2020), bioactive glass (Ojansivu et al., 2019) and silicate nanoplatelets (Byambaa et al., 2017). To further promote vascularization, human umbilical vein endothelial cells (HUVECs) have been encapsulated into GelMA hydrogel bioinks to engineer vasculogenic niches. Moreover, to promote vascular spreading, chemically conjugated VEGF were introduced in the surrounding bone niches (Byambaa et al., 2017).

Although extrusion bioprinting is a common and accessible bioprinting technology compatible with a large variety of bioinks, other bioprinting technologies have been developed to overcome the main limitations of extrusion-based printing including lithography bioprinting and spheroid bioprinting (Daly et al., 2021b). Lithography bioprinting technology can create physical features at the scale of 10–100 μm, which is a significant advantage over extrusion bioprinting in which the minimum resolution is ∼100 μm (Bertlein et al., 2017; Lim et al., 2018). Spheroid bioprinting, which processes self-organized tissues (often cell spheroids) into 3D constructs to scale and direct self-organization, can mimic tissue-like features and achieve high cell densities to promote cell–cell contacts (Skylar-Scott et al., 2019; Daly et al., 2021a). There are numerous interesting and promising applications of lithography and spheroid bioprinting techniques to fabricate complicated *in vitro* systems that would otherwise be challenging for extrusion-based processes to realize, including a liver lobule model (Ma et al., 2016), alveolar lung model (Grigoryan et al., 2019), and other organ and tissue models (Grigoryan et al., 2019; Daly et al., 2021a). Until now, dental, oral, and craniofacial applications using these novel bioprinting technologies for repair and regeneration have been scarce.

## Clinical Applications for Dental Regenerative Medicine

### Personalized Reconstruction With Image-Based Scaffolds

The numerous bones of the craniofacial skeleton exhibit variable anatomical forms and exist in intimate relation to one another, as well as to abundant nerves and vessels. As such, bony reconstruction within this region often entails labor-intensive, multi-step operations with limited surgical access to morphologically complex defects. In the early stage of AM, stereolithographic models were introduced as an adjunct to standard diagnostic imaging and casts. These 3D models improved surgeon visualization of bony defects and their spatial relationship to adjacent structures, thus enhancing accuracy in preoperative evaluation, diagnosis, and treatment planning (D’Urso et al., 1999). With significant advancements in high resolution medical imaging and CAD-CAM software, AM processes are now employed to fabricate personalized constructs for a vast range of applications in all phases of craniomaxillofacial surgery (Levine et al., 2012; Yu et al., 2019).

In a recent systematic review of customized objects used in 3D printing-assisted craniofacial and maxillofacial operations, four major categories of personalized constructs were identified: (1) contour models; (2) guides; (3) splints; and (4) implants (Jacobs and Lin, 2017). Contour models facilitate accurate prebending of reconstruction meshes or plates, eliminating the need for extensive intraoperative manipulation and significantly reducing operating time (Sumida et al., 2015; von Wilmowsky et al., 2020). Guides utilize negative space relative to patient anatomy to provide intraoperative reference for precise osteotomy preparation and controlled positioning of dental (Ersoy et al., 2008) and zygomatic (Wang et al., 2020) implants. Splints are similar to guides; however, they are fabricated to align structures in virtually planned, postoperative positions. Finally, implants are medical devices surgically placed patient tissues. Customized CAD-CAM devices have been employed for human clinical use in the reconstruction of structures such as the temporomandibular joint (Ackland et al., 2018), maxilla and mandible (Lethaus et al., 2012; Ma et al., 2017; Chiapasco et al., 2021), paranasal sinuses, nasal bone (Horn et al., 2012), orbit (Bachelet et al., 2018), and cranial vault (Jardini et al., 2014; Park et al., 2016; Unterhofer et al., 2017). Use of components produced by AM can also minimize discrepancies between planned and actual surgical outcomes. For example, in a case series documenting nine patients undergoing orthognathic surgery or distraction osteogenesis procedures, the use of custom templates and reconstruction microplates enabled accurate repositioning of the maxillary segment within 1 mm of the digitally planned centroid position and 1° orientation in all linear and axial directions, respectively (He et al., 2015).

Additive manufacturing is now widely employed in dentistry for a variety of purposes, including fabrication of dentures, occlusal splints, temporary crowns and bridges, orthodontic appliances, and surgical guides. More recently, customized, non-resorbable titanium metal cages intended for extraosseous alveolar ridge augmentation have become available to clinicians as well. Despite prolific use of commercially available AM equipment to create custom dental devices in every day clinical practice, attempts to utilize bioresorbable bone scaffolds produced by this technology have only recently begun in academic, clinical settings. In 2015, the first dental use of a personalized, bioresorbable scaffold produced with AM in humans was reported. A PCL scaffold fabricated by selective laser sintering was loaded with rhPDGF-BB solution and implanted into a periosseous defect in the periodontium localized to a mandibular canine site (Rasperini et al., 2015). The design incorporated novel, cylindrical-shaped, PDL fiber-guiding architecture previously reported in a rodent model (Park et al., 2012). At 1 year, a modest 3 mm gain in clinical attachment and partial root coverage was achieved but graft exposure culminated in scaffold failure (Rasperini et al., 2015). This complication also occurred in a case series assessing the use of prefabricated, FDM-printed, PCL scaffolds for alveolar ridge preservation (Goh et al., 2015). The scaffolds remained largely intact within the healing extraction sockets for 6 months and 2/13 patients experienced manageable graft exposure, highlighting the challenges posed by the slow resorption rate of PCL.

Customized, bioresorbable bone scaffolds created by AM processes have been tested more extensively in the fields of maxillofacial and craniofacial surgery. In 2016, a clinical case series described 20 patients who received artificial bone constructed with 3D inkjet printing to graft non-weight bearing, maxillofacial bone deformities in 23 sites (Saijo et al., 2009; Kanno et al., 2016). These scaffolds were fabricated in 0.1 mm layers by spraying hardening liquid composed of 5% sodium chondroitin sulfate, 12% disodium succinate, and 83% distilled water onto an α-TCP powder. At 1 year, 18 of 21 remaining grafted sites demonstrated CT values indicative of satisfactory bone union. Bacterial infection necessitated removal in 4 sites within a period of 1 – 5 years postoperatively. Failures tended to occur in grafts spanning larger missing bone volume or in one case, a patient that was a carrier of MRSA. With the longest follow-up period occurring just over 7 years, none of the artificial bones demonstrated complete replacement and only partial new bone formation was observed within the scaffold. Despite the limited success observed in these initial translational studies, these efforts represent the emergence of image-based, bioresorbable scaffold technology in the clinical arenas of dental and craniomaxillofacial surgery.

### Clinical Impact

Bone grafting is a routine procedure in clinical dentistry and may occur in approximately half of all dental implant sites (Cha et al., 2016). Augmentation of the alveolar ridge through procedures such as guided bone regeneration and maxillary sinus lifts, are often necessary to create adequate bone volume prior to implant placement. Due to a high frequency of bone grafting procedures in the healthcare field overall and a limited pool of musculoskeletal tissue donors, increased use of bone graft substitutes relative to autogenous grafts is a precipitating trend (Kinaci et al., 2014). This is reflected in the global market for dental bone graft substitutes, which had an estimated value of $450 million in 2020 and is projected to reach $659 million by 2025 (Markets and Markets, 2020). In the future, AM may yield a new generation of bone graft substitutes that achieve improved regenerative outcomes by uniting the versatility of CAD-CAM technology with modern tissue engineering principles and a personalized medicine treatment approach.

Contemporary biomedical research is steadily approaching a reliable AM strategy to create bioresorbable bone scaffolds for clinical use and the implications for patient care are enormous. Recently, a workflow for AM fabrication of porous, bioresorbable scaffolds consisting of medical grade PCL for the reconstruction of large, posterior mandibular defects was demonstrated (Bartnikowski et al., 2020). The resultant porosity (83.91%) and mean pore size (590 ± 243 μm) were within suitable ranges for bone regeneration and the mean discrepancy between the template implant model and the scanned scaffold was found to be 74 ± 14 μm, representing a level of accuracy adequate for clinical application. Pending further preclinical validation and clinical trials, rapid in-house fabrication and deployment of personalized bone scaffolds with accurate replication of individual patient anatomy could revolutionize trauma care in the fields of maxillofacial and craniofacial surgery. Esthetics, form, and function could also be restored in patients that have undergone massive tumor resection in craniofacial structures. In implant dentistry, substantial augmentation or reconstruction of the alveolar ridge could be accomplished with personalized constructs rather than adapting universal materials to anatomically diverse defects. Invasive procedures, such as autogenous harvesting of large, block grafts from secondary surgical sites or the placement of zygomatic implants, which are reserved for severely atrophic maxillae, may also be avoided. Finally, AM offers new strategies to employ scaffolds as carriers for exogenous agents that not only enhance regeneration but offer therapeutic benefit to the patient as well. This may be especially valuable for modulation of the destructive, biochemical mechanisms inherent to tissues affected by chronic inflammation, such as in periodontal disease.

## Future Directions

### Challenges in Additive Manufacturing

AM holds tremendous promise for the advancement of regenerative medicine; however, this impressive technology must overcome several obstacles before it can be extensively introduced to clinical settings for the purpose of fabricating personalized bone tissue scaffolds. First, AM has historically been limited by relatively low production speed. In 2017, the average build time to create a personalized object constructed by AM techniques in the field of craniomaxillofacial surgery is approximately 18.9 h but can be as high as 96 h per object (Jacobs and Lin, 2017). Practical utilization requires faster manufacturing processes that maintain adequate print resolution, surface quality, and mechanical integrity, especially to hold relevance for applications in urgent care. Use of modern multi-extrusion printing systems is swiftly rising in tissue engineering for bone and periodontal structures due to its one-step printing approach, improved speeds, and ability to use versatile material formulations (Porta et al., 2020). Second, variations in part quality can occur due to errors introduced during the digital manipulation of virtual models or during the physical construction process. Third, decentralization of bioresorbable scaffold fabrication from commercial biomaterial manufacturing facilities to local centers of production may complicate safety assessments and reporting of adverse events. Last, the use of customized scaffolds with innumerable variations in composition and design may present significant challenges for standardized regulation in the clinical dental practice setting. Development and oversight of appropriate guidelines for post-manufacturing quality assurance and sterilization would be required. In the case of implantable scaffolds, sterilization is especially critical to prevent infection. Scaffolds constructed with certain polymeric materials may not be able to withstand the high temperatures necessary for autoclave sterilization and would require alternative sterilization methods.

Despite these potential limitations, the technologic infrastructure necessary to produce bioresorbable scaffolds for bone regeneration by AM is available for implementation. Successful translation to clinical use now relies upon the ability to manipulate biomaterials and precisely coordinate their architectural and biochemical features with known physiologic mechanisms of tissue formation. This process is further complicated by the addition of exogenous agents, such as viable cells, growth factors, gene vectors, drugs, and other bioactive components, which must be present in appropriate quantities to simulate a suitable microenvironment for regeneration. Simultaneous incorporation of viable cells during scaffold fabrication remains a preeminent challenge. Ensuring cell survival during the AM process and preserving their phenotype and morphology post-processing will require continued development of cell-deposition techniques and cell-carrier systems. Improved control of cellular responses will benefit from the progressive advancement of scaffold-based gene therapy techniques. Finally, innovation in the use of hydrogel bioinks and 3D bioprinting processes will further refine spatiotemporal regulation of biomolecular signaling and progress efforts to regenerate bone tissue with bioresorbable, biomimetic scaffolds *in vivo*.

### Emerging Additive Manufacturing Research With Potential for Dentistry

A successful, alloplastic bone substitute biomaterial fabricated by AM is a treatment concept just on the horizon of realistic clinical practice and there are many exciting implications for the future. AM has given rise to a new manufacturing concept termed four-dimensional (4D) printing, in which time is the fourth dimension of the printed construct (Saska et al., 2021). 4D printing aims to create scaffolds fabricated with advanced or “smart” materials that react to external stimuli such as pH, humidity, light, and temperature, allowing dynamic responses to *in vivo* conditions (Valvez et al., 2021). Potential applications of these sensitive materials include utilizing environmental stimuli to induce appropriate release patterns of angiogenic and osteogenic factors during wound healing and tissue formation processes, thus enhancing regenerative capability. Even further, shape-morphing (Gladman et al., 2016) and shape-memory materials (Zhou and Sheiko, 2016; Liu et al., 2020) are setting the stage for scaffold materials capable of controlled self-assembly or even self-repair (Zhang et al., 2019), which may offer pivotal advantages for the regeneration of weight-bearing structures, such as the jaws or temporomandibular joint. Lastly, *in situ* bioprinting, which entails real-time scaffold fabrication directly within the defect (O’Connell et al., 2016; Di Bella et al., 2018), is an interesting development of AM technology with clinical potential for bone regeneration (Keriquel et al., 2010; Keriquel et al., 2017), especially in defect sites with complex morphologies, significant undercuts, or limited surgical access; examples of potential applications include bone grafting of the maxillary sinuses, intrabony defects, and peri-implant defects.

## Conclusion

The treatment of dental, oral, and craniofacial bone defects is currently restricted by available biomaterials, which have limited capacity to facilitate true regeneration of new tissues that exhibit native physiologic form, function and esthetics. Further research efforts are needed to optimize AM for the production of bioresorbable scaffolds that yield safe, predictable, and efficacious clinical outcomes in the reconstruction of bony defects. More preclinical studies are needed to improve the material properties and clinical performance of polymer-ceramic composite scaffolds for bone reconstruction and to refine understanding of the architectural features that promote formation of an anatomic periodontal ligament compartment. Additionally, tremendous opportunity exists to functionalize scaffolds for therapeutic purposes, especially with regards to gene therapy. As the understanding of multifaceted biomaterial interactions and tissue dynamics improves within the scientific community, AM offers a promising future in which a superior generation of sustainable regenerative biomaterials will become accessible for everyday clinical use. Commercialization of custom scaffold technology will dramatically accelerate the trend toward increased usage of synthetic bone substitutes and expand their existing market share within the multibillion dollar industry for biomaterials. Successful adaptation of AM technology for bone tissue engineering will expose a new realm of regenerative possibilities within dental medicine, thus expanding treatment options for patients and significantly improving their oral health related quality-of-life. Eventually, personalized bone constructs for dental regenerative medicine will evolve from state-of-the-art technology to a new standard in patient care.

## Author Contributions

WG and JL contributed to conception and design. JL led the writing of the manuscript. SM, YY, DW, and MC wrote sections of the manuscript. All authors contributed to manuscript revision. All authors read and approved the submitted version.

## Conflict of Interest

The authors declare that the research was conducted in the absence of any commercial or financial relationships that could be construed as a potential conflict of interest.

## Publisher’s Note

All claims expressed in this article are solely those of the authors and do not necessarily represent those of their affiliated organizations, or those of the publisher, the editors and the reviewers. Any product that may be evaluated in this article, or claim that may be made by its manufacturer, is not guaranteed or endorsed by the publisher.
